# Mixed comparison of different exercise interventions on physical functioning in adult patients with morbid obesity following bariatric surgery: a systematic review and network meta-analysis

**DOI:** 10.3389/fendo.2024.1465718

**Published:** 2024-10-18

**Authors:** Chen Hu, Dong Sun, Yufei Fang, Xuanzhen Cen, Yining Xu, Julien S. Baker, Yaodong Gu

**Affiliations:** ^1^ Ningbo No. 2 Hospital, Ningbo, China; ^2^ Faculty of Sports Science, Ningbo University, Ningbo, China; ^3^ Department of Sport and Physical Education, Hong Kong Baptist University, Hong Kong, Hong Kong SAR, China

**Keywords:** morbid obesity, bariatric surgery, exercise interventions, physical functioning, network meta-analysis

## Abstract

**Introduction:**

People who are overweight following bariatric surgery (BS) often need physical exercise to help with body function. However, it is not known which exercise interventions are more effective in improving physical function.

**Methods:**

PubMed, Web of Science, Embase, and Cochrane Library databases were systematically searched for randomized controlled trials evaluating the effects of different exercise interventions on physical function in patients with excessive obesity following bariatric surgery. Outcome measures included effect sizes for physical function (PF), expressed as the number of stand-sit passes or the maximum distance walked within a time limit, body mass index (BMI), and blood pressure (BP). A systematic review was conducted to screen and synthesize the included studies, followed by a network meta-analysis for quantitative data analysis.

**Results:**

A total of 15 studies involving 1011 patients were included. For PF, telehealth core exercise had the highest probability (0.46) of being the most effective intervention. For BMI, nutritional behavior and guided exercise, intervention had the highest probability (0.27) of being the most effective. Regarding BP, exercise prescription had the highest probability (0.47) for improving systolic blood pressure, while aerobic and strength and flexibility training had the highest probability (0.6) for improving diastolic blood pressure.

**Discussion:**

Telehealth core exercise may be the most effective intervention for enhancing PF in overweight patients after bariatric surgery. Changes in BMI and BP with different postoperative exercise interventions may depend more on the surgery itself than the exercise modality. More specifically designed RCTs are needed for reliable conclusions.

**Systematic review registration:**

https://www.crd.york.ac.uk/prospero, identifier CRD42024507209.

## 1 Introduction

Overweight and obesity, as defined by the World Health Organization (WHO), constitute health risks resulting from abnormal or excessive fat accumulation (1). These conditions are associated with a heightened risk of severe health issues, including heart disease, stroke, type 2 diabetes, and certain cancers (2). Obesity-related factors contribute to over 2.8 million premature deaths annually, making obesity the fifth leading cause of premature death globally.

Bariatric surgery (BS) is often regarded as a non-conservative treatment option when standard weight loss methods, such as lifestyle changes, prove ineffective (3). Surgical treatment for obesity is substantially more effective than traditional approaches, yielding significant and sustained weight loss while alleviating or resolving obesity-related complications. However, BS is not without side effects. Research indicates that approximately 30% of weight loss following BS can be attributed to a reduction in muscle mass, potentially impairing physical mobility and muscle strength (4–8). Consequently, numerous studies have advocated for exercise interventions post-BS to mitigate or eliminate these adverse effects through physical activity (53).

Caspersen, Powell, and Christenson defined physical activity as “any bodily movement produced by skeletal muscles that results in the expenditure of energy.” The term “exercise” is further specified as “planned, organized, repetitive, and purposeful movement intended to improve or maintain one or more components of physical fitness” (9). Even modest increases in physical activity (PA) have been shown to impact morbidity and mortality significantly. For example, individuals engaging in 15 minutes of physical activity daily can reduce their risk of all-cause mortality by 14% and extend their life expectancy by three years compared to those who remain inactive (10). Regular exercise can also lower blood pressure in 75% of people with hypertension, with average systolic and diastolic reductions of 11 and 8 mm Hg, respectively (11). Additionally, each extra 15 minutes of activity beyond the initial 15 minutes per day further reduces all-cause mortality by 4% and all-cancer mortality by 1%. These benefits are observed across various age groups and genders and in individuals at high risk for cardiovascular disease. Engaging in four hours of light-intensity PA per week has been shown to decrease all-cause mortality by 38% compared to sedentary behavior. Furthermore, each additional hour of PA per week is significantly associated with increased survival. It is suggested that an increase of 15 minutes per day (equivalent to 105 minutes per week) may be considered a clinically significant improvement (12).

In recent years, there has been increasing interest in the effects of various exercise modalities on obese patients’ post-bariatric surgery (OPBS). These studies can be categorized into three main groups. The first group comprises studies focusing on exercise training, aiming to investigate the effects of endurance training, strength training, and other physical fitness regimens on OPBS (13–22). The second group includes studies conducted from a nutritional perspective, examining the interaction between protein and other nutrients with physical activity in this population (23, 24). The third group consists of studies from a health management perspective, exploring the impact of different exercise prescription on OPBS (25–27). Despite the diversity of exercise interventions studied, no trials have comprehensively compared these interventions, making it challenging to determine the most effective approach for improving physical function in OPBS.

In light of the above, this study employs a network meta-analysis to identify the most effective exercise intervention for enhancing physical function in OPBS. This analysis comprehensively compares the effects of various exercise interventions on their physical function.

This systematic review and meta-analysis adhered to the PICOS framework, which includes defining participants (P), interventions (I), comparisons (C), outcomes (O), and study design (S). Each of these elements was rigorously applied to ensure a structured and comprehensive evaluation of the available evidence.

## 2 Materials and methods

### 2.1 Protocol and registration

This review adhered to the guidelines outlined in the Cochrane Handbook and the Preferred Reporting Items for Systematic Reviews and Meta-Analyses (PRISMA) statement (28), (29). The review protocol was available on PROSPERO (https://www.crd.york.ac.uk/prospero) and the registration number for this systematic review was CRD42024507209.

### 2.2 Eligibility criteria

#### 2.2.1 Population

The population under investigation in this systematic review was defined as follows: (1) participants were aged between 18 and 60 years; (2) participants had undergone bariatric surgery; (3) participants had a body mass index (BMI) of 30 kg/m² or higher.

#### 2.2.2 Interventions

Due to the influence of COVID-19, there were large differences in exercise intensity and duration of intervention and follow-up between intervention trials, and it was difficult to assess the effects of interventions more accurately by relying only on relatively broad groupings from the training, diet and care perspectives; therefore, this systematic review divided the included interventions relatively independently and included the following intervention trial studies: (1) Aerobic and strength and flexibility training, with the abbreviation being “ASFT”; (2) Aerobic and strength training, with the abbreviation being “AST”; (3) Aerobic training, with the abbreviation being “AT”; (4) Exercise prescription, with the abbreviation being “EP”, which includes three core individualized elements: dialogue, recommendation of physical activity with a written prescription, and follow-up; (5) Moderate-intensity aerobic exercise, with the abbreviation being “MAE”; (6) Nutritional behavior and guidance exercise interventions, with the abbreviation being “NBGEI”; (7) Protein intake intervention, with the abbreviation being “PII”; (8) Protein intake, and supervised strength training, with the abbreviation being “PISST”; (9) Transtheoretical model–based exercise training program, with the abbreviation being “TMET”; (10) Telehealth core exercise, with the abbreviation being “TCE”.

#### 2.2.3 Comparators

The systematic review included trials in which the participants were asked to receive standard care, including regular medical follow-up, with no additional exercise intervention. The aforementioned trials were designated as the comparators and were abbreviated as “Control.” All of the aforementioned interventions could also be regarded as comparators.

#### 2.2.4 Outcomes

The primary outcome was physical function, which was measured by either the 6/12-minute walk test or the 30/60-second stand-and-sit test (17, 30). Secondary outcomes included body mass and blood pressure, which were measured using the body mass index and systolic and diastolic blood pressures. All three of these variables were presented as mean ± standard deviation.

#### 2.2.5 Study

This systematic review included only studies of randomized controlled trials (RCTs). All studies that met the eligibility criteria were required to be published in peer-reviewed English-language journals.

#### 2.2.6 Exclusion criteria

Exclusion criteria for study were: (1) the type of literature was a review, conference literature or case study; (2) non-English language literature; (3) animal experiments; and (4) the research literature lacked a clear statement of the relevant baseline or outcome indicators.

### 2.3 Information sources

The Web of Science, Pubmed, Embase and Cochrane Library databases were searched electronically from inception to 20 January 2024, with the language restriction of English. In addition, the ClinicalTrials.gov website was searched for unpublished data. We also reviewed the references of identified records to identify relevant articles.

### 2.4 Search

Search terms included the keywords and corresponding MeSH terms for bariatric surgery, gastrectomy, gastric bypass, biliopancreatic diversion, gastroplasty, morbid obesity, body mass index, exercise, walking, training, physical activity, and randomised controlled trials. The complete list of search terms was provided in **Supplementary Data Sheet 1** for reference.

### 2.5 Study selection

The screening process for eligible studies involved thorough examination of titles, abstracts, and full texts to ensure comprehensive inclusion in this systematic review. Initially, all retrieved studies were imported into EndNote for screening and duplicate removal. In the first stage, two independent authors scrutinized the titles, followed by a secondary review of abstracts by another pair of authors in the subsequent stage. Finally, a third independent author assessed the full texts of studies excluded in the second stage to determine their suitability for inclusion in the systematic review.

### 2.6 Data collection process

All the original data were extracted independently by two authors. Checking and confirming of extracted data by a third independent author.

### 2.7 Data items

To facilitate comparison of the aggregated effects of various interventions, we meticulously gathered trial details from each study, encompassing population demographics such as average age and gender distribution, along with intervention protocols categorized accordingly. These particulars were then synthesized and documented in an extraction sheet. The outcomes of this process were essential for assessing inter-study heterogeneity, potential biases, and establishing a foundation for the pooled evidence.

For the network meta-analysis, trial data was meticulously recorded in a separate extraction sheet to facilitate data preprocessing. This entailed capturing sample size (N), mean values (Mean), and standard deviations (SD) of each outcome at baseline and subsequent recording points. These metrics were imperative for detecting inconsistencies.

### 2.8 Risk of bias within Individual Studies

The risk of bias within individual studies was assessed utilizing the Cochrane Collaboration Risk of Bias Assessment Tool within the Cochrane Library Review Manager software (Version 5.3, Wiley, Chichester, United Kingdom) by two independent assessors (31). In cases of discrepancy, an impartial arbitrator was engaged to adjudicate.

### 2.9 Summary measures

The process of data extraction and integration was undertaken by two independent authors utilizing Microsoft Office Excel (https://www.microsoftstore.com). The outcomes related to physical function were delineated as standardized mean difference and standard error, while those associated with BMI and blood pressure were expressed as mean difference and standard error. Subsequently, a network meta-analysis was performed on the integrated data using the Aggregate Data Drug Information System (ADDIS) software (http://www.drugis.org/addis).

### 2.10 Assessment of inconsistency

The Aggregate Data Drug Information System (ADDIS) software was employed for data summarization and analysis. To determine consistency, the first step was to check for a closed loop of evidence in the evidence graph. If such a loop existed, it was necessary to compare the standard deviations under both consistency and inconsistency assumptions and ensure that all p-values in the node-splitting analysis were greater than 0 (32). This was because, when outcomes were interpretable, it was necessary to distinguish between models for each split node (33). If there was no closed loop, simply compared the standard deviations under the consistency and inconsistency assumptions. If these conditions were met, the consistency model could be selected (34).

In the consistency model, outcomes were depicted through a rank probability plot, where the cumulative sum of all rank probabilities equaled 1 (35). For PF, higher rank numbers in the plot denoted greater improvements, whereas for BMI, SBP, and DBP, lower rank numbers indicated greater improvements (13). Following the determination of the data analysis model and subsequent reporting of results, a league table was provided, illustrating the standard mean difference between column-defining and row-defining items (36). In the context of the inconsistent model, outcomes were presented in tabular format.

### 2.11 Risk of bias across Studies

Two independent evaluators employed the Cochrane Collaboration Risk of Bias Assessment Tool within the Cochrane Library Review Manager software version 5.3 to appraise the risk of bias across studies (37).

## 3 Results

### 3.1 Study selection

**Figure 1:**
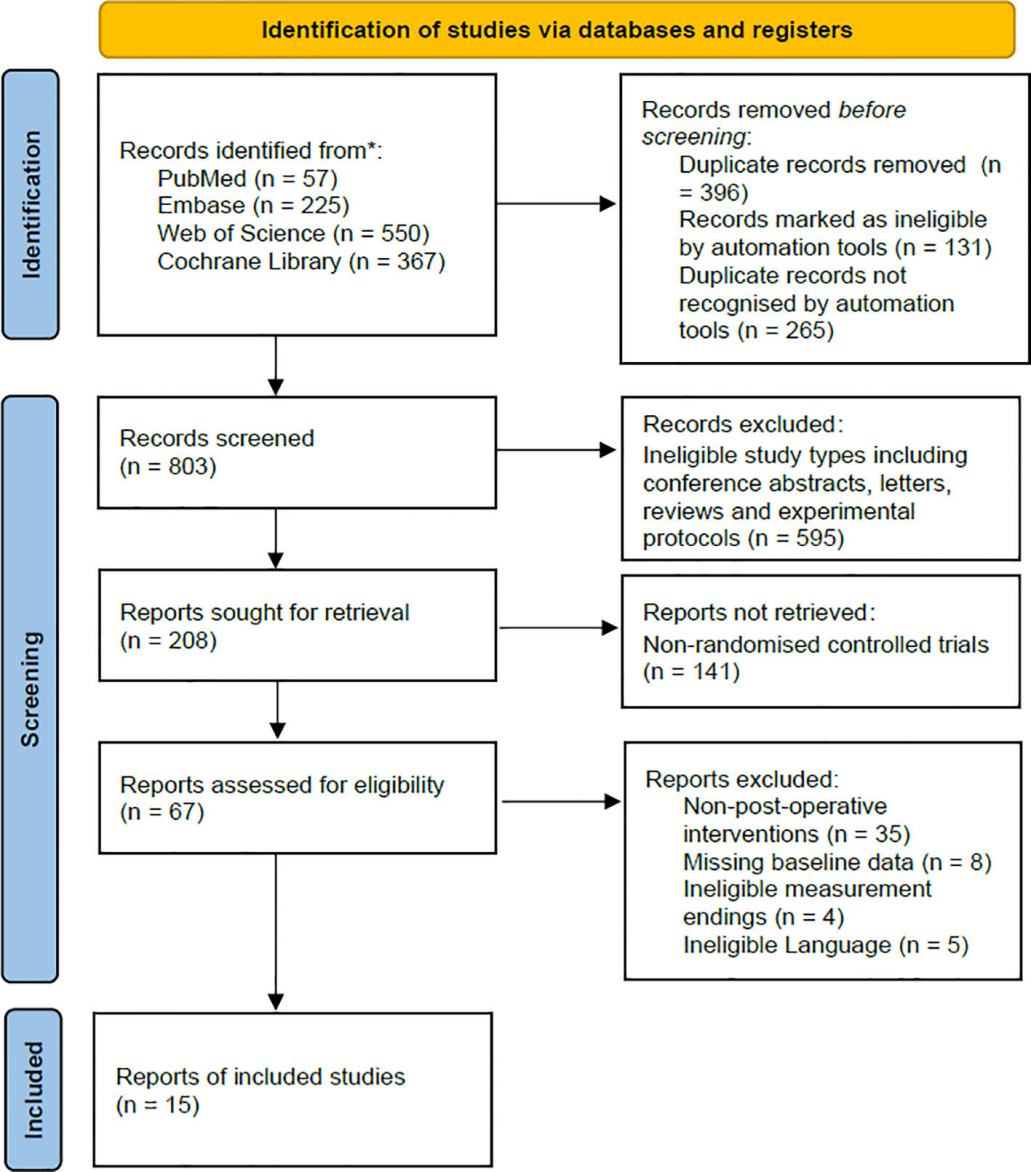
Flow diagram of the study selection.

A total of 1,199 titles and abstracts underwent initial screening. Following the removal of 396 duplicates, 803 studies proceeded to the record screening stage. During this process, 595 non-published experimental reports and 141 non-RCT reports were excluded. Subsequently, 67 reports underwent full-text screening. Among these, 47 reports were excluded due to intervention criteria not being met, and 5 reports were excluded due to language limitations. Ultimately, 15 reports were deemed suitable for inclusion in the systematic review (13–27). The flow chart depicting this process was presented in **Figure 1**.

### 3.2 Study characteristics

Among the 15 studies included, 12 investigated the impact of various exercise interventions on BMI among individuals with excessive obesity following bariatric surgery. Six studies examined the effects of different interventions on BP, while 9 trials assessed the impact of various interventions on PF.

### 3.3 Geometry of network

**Figure 2:**
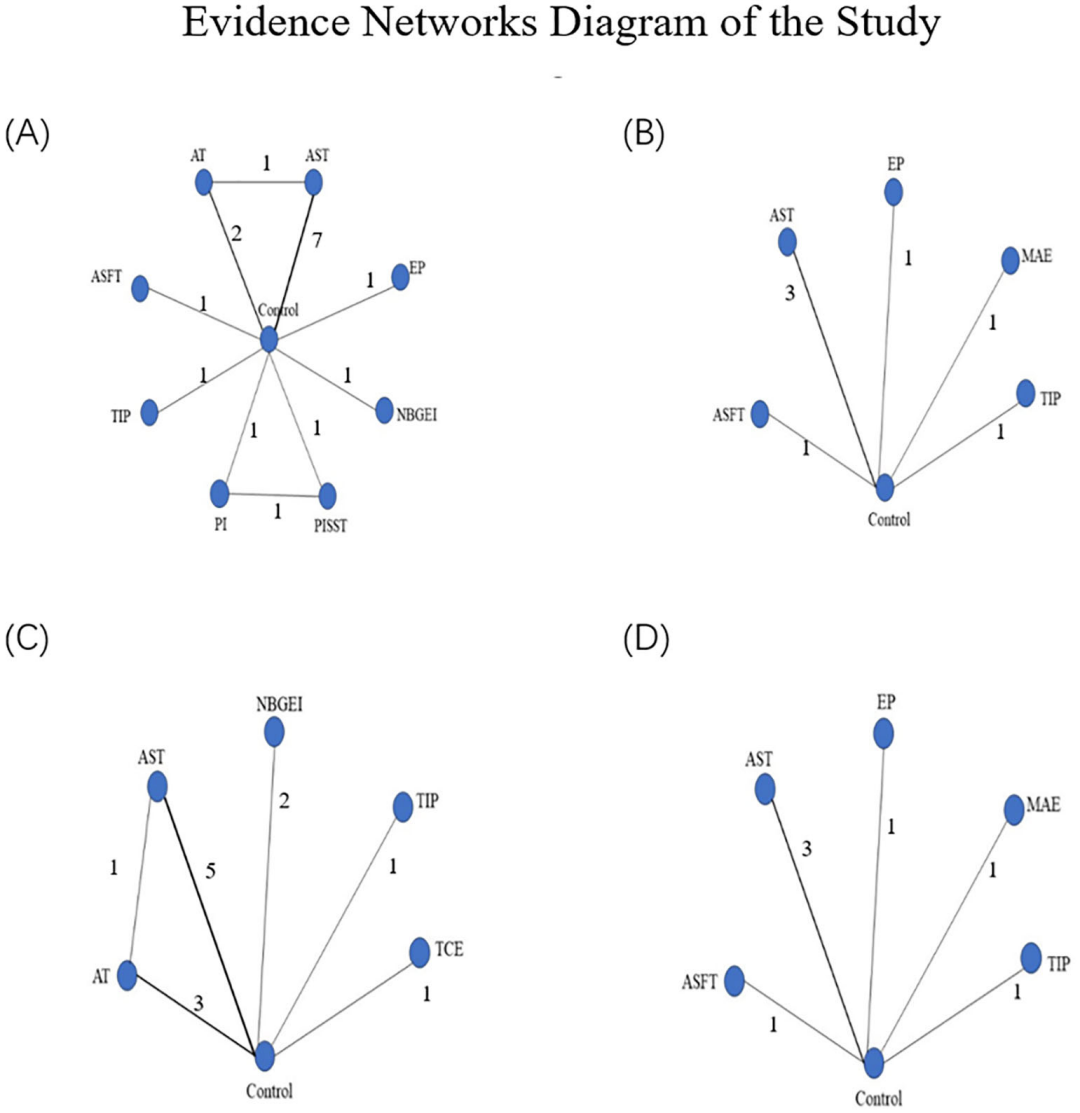
**(A)** Presented the network of evidence for BMI; **(B)** presented the network of evidence for DBP; **(C)** presented the network of evidence for PF; **(D)** presented the network of evidence for SBP. Evidence networks diagram of the study: ASFT, Aerobic and strength and flexibility training; AST, Aerobic and strength training; AT, Aerobic training; EP, Exercise prescription; MAE, Moderate-intensity aerobic exercise; NBGEI, Nutritional Behavior and Guidance Exercise Interventions; PI, Protein intake group; PISST, Protein intake, and supervised strength training group; TIP, TTM-based intervention program; TCE, Telehealth core exercise.

As depicted in **Figure 2**, network geometry visually illustrated the evidence structure. Each intervention was represented by a node, with direct comparisons between pairs of interventions indicated by edges (38). The number on each edge denoted the number of arms in each comparison. **Figures 2A**–**D** depicted the evidence plots for BMI, diastolic blood pressure, physical functioning, and systolic blood pressure, respectively.

### 3.4 Risk of bias within studies

**Figure 3:**
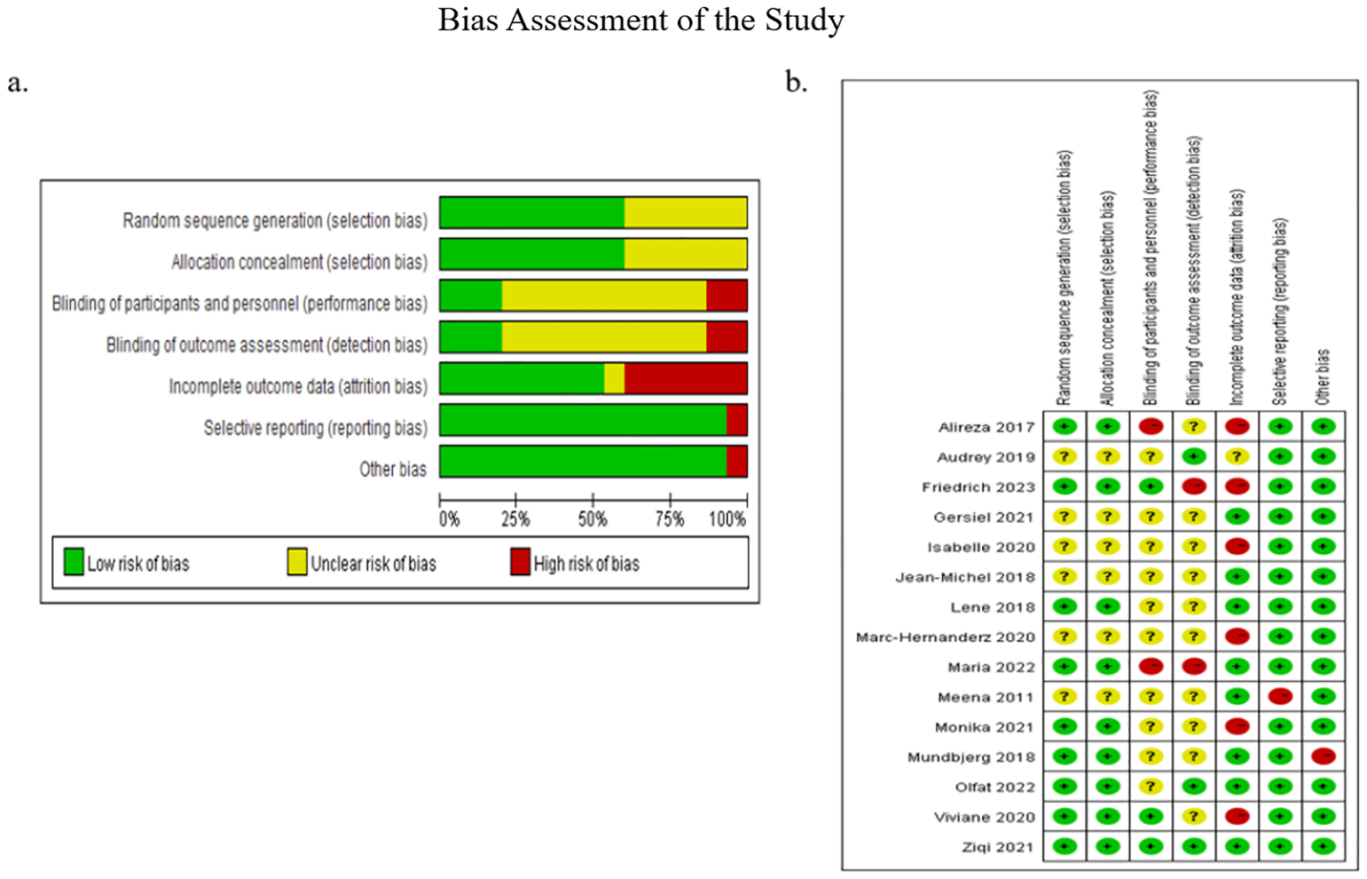
Bias assessment of the study. **(A)** Illustrated the overall bias; **(B)** depicted the collective outcomes.

**Figure 3**. presented the results of the risk of bias assessment. Following a discussion, a consensus was reached on all items. The overall bias was illustrated in **Figure 3A**. Firstly, the risk of selection bias (random sequence generation and allocation concealment) was low, with 9 studies identified as having a low risk of bias in this area. Secondly, the risk of performance bias (blinding of participants and personnel) and the risk of detection bias (blinding of outcome assessment) were considered to be of a medium level of concern (unclear in 10 studies). Thirdly, the risk of attrition bias (incomplete outcome data) was low (low in 8 studies), and the risk of reporting bias (selective reporting of outcomes) and other bias were low (low in 14 studies). **Figure 3B**, which depicted the collective outcomes, revealed that three studies exhibited a considerable degree of bias, six studies exhibited a moderate degree of bias, and six studies exhibited a minimal degree of bias.

### 3.5 Results of individual studies

**Table 1** showed the characteristics of the included trials and all original data were available in the **Supplementary Data Sheet 1**. In the final 15 included studies, the mean age of the patients ranged from 25.0 to 53.9 years. Of these, 450 patients served as controls and only received routine postoperative care.

**Table 1:** Characteristics of the included trials.

Study	Population	Sex	Surgery Included	Interventions	Main Findings
(16)	Obese women, BMI ≥ 40 kg/m2, undergoing a Roux-en-Y GBS for more than 5 years.	Only women.	Roux-en-Y GBS.	Aerobic training.	Morbidly obese women have slower HR kinetics and altered cardiac modulation during submaximal exercise. However, aerobic exercise training can produce beneficial adaptations in HRV and faster HR kinetics following GBS.
				Standard care for OPBS.	
(23)	Adults aged 18 to 65 years, planning to undergo either primary gastric bypass or sleeve gastrectomy.	Male 33(21.6%), Female 120 (78.4%).	Primary gastric bypass or sleeve gastrectomy surgery.	Nutritional counselling and exercise guidance.	A lifestyle intervention in the first 12 months following bariatric surgery does not improve weight loss or health outcomes.
				Standard care for OPBS.	
(26)	Patients slated for laparoscopic Roux-en-Y gastric bypass surgery over 30 months.	49 female (86%) in intervention group, 47 female (73%) in control group.	Laparoscopic Roux-en-Y gastric bypass.	Exercise prescription.	Patients in both groups lost considerable weight, had reduced waist circumference, and increased PA. Although marked differences between groups were not observed over one year, the intervention group increased its PA 6-months postoperatively.
				Standard care for OPBS.	
(15)	Participants at least 6 months after bariatric surgery.	35 females and 19 males.	Bariatric surgery.	Telehealth supervised home core stabilization program.	Eight weeks of a home-based telehealth core exercise program improves core endurance, postural stability, and aerobic capacity in patients following BS.
				Standard care for OPBS.	
(14)	Patients aged between 18–60 years old with no absolute contraindications to practice physical exercise.	21 were allocated to intervention group (6 men and 15 women) and 22 were allocated to control group. (5 men and 17 women).	Sleeve gastrectomy.	Aerobic and strength and flexibility training.	Physical exercise in obese patients undergoing bariatric surgery increased functional capacity independently of weight losses resulting from bariatric surgery.
				Standard care for OPBS.	
(27)	Obese adults age ≥ 18 years, body mass index ≥ 28.0 kg/m2, underwent initial Roux-en-Y gastric bypass, sleeve gastrectomy, or jejunojejunal bypass.	60 were allocated to intervention group (16 men and 44 women) and 60 were allocated to control (25 men and 35 women).	Initial Roux-en-Y gastric bypass, sleeve gastrectomy (SG), or SG + jejunojejunal bypass.	Transtheoretical model–based exercise training program	Compared with the control group, the 12-week TTM-based intervention significantly helped participants advance, perceive more benefits and fewer barriers to exercise, and show higher exercise adherence and better physical function afterward.
				Standard care for OPBS.	
(25)	People had Roux-en-Y gastric bypass (n = 50) and sleeve gastrectomy (n = 17) within the past 12 months and had not been presently involved in exercise training.	33 were allocated to intervention group (5 men and 28 women) and 37 were allocated to control group (6 men and 31 women).	Roux-en-Y gastric bypass and sleeve gastrectomy.	Aerobic and strength training.	The intervention was overall ineffective, except for slight, but significant improvements in selected functional outcomes.
				Standard care for OPBS.	
(13)	Severely obese patients planned to undergo biliopancreatic diversion with duodenal switch (n=30) or sleeve gastrectomy (n=30). All of them were between 18 and 65 years of age, <200 kg.	Male 13(22%), Female 45 (78%).	Duodenal switch or sleeve gastrectomy.	Aerobic and strength training.	Twelve weeks of supervised aerobic and resistance training program initiated 3 months after bariatric surgery has an added benefit on improving cardiorespiratory fitness and preserves leg muscle mass in severely obese patients.
				Standard care for OPBS.	
(22)	Sixty severely obese men and women (biliopancreatic diversion with duodenal switch = 30, sleeve gastrectomy = 30), aged between 18 and 65 years.	34 were allocated to intervention group (8 men and 26 women) and 15 were allocated to control group (4 men and 11 women).	Sleeve gastrectomy and biliopancreatic diversion with duodenal switch.	Aerobic and strength training.	Similar weight loss on HDL-cholesterol levels without additional effect on LDL-cholesterol levels.
				Standard care for OPBS.	
(18)	21 patients underwent sleeve gastrectomy after a 3-year follow-up.	Intervention group, n=10(male 1, female 7); control group, n=8(male 3, female 7).	Sleeve gastrectomy.	Aerobic and strength training.	In the medium-term after sleeve gastrectomy exercise may contribute to prevent weight regain and to reduce fat mass, glycaemia, and blood cholesterol.
				Standard care for OPBS.	
(24)	Female between 18 and 65 years, and BMI of 40 kg/m2 or higher or BMI of 35 kg/m2 or higher with at least one obesity comorbidity.	76 women, no men.	Roux-en-Y gastric bypass.	Additional oral protein intake and supervised resistance training.	Loss over time in lean body mass did not differ between groups. Loss in muscle strength observed after bariatric surgery can be overcome by resistance training with additional protein intake.
				Additional oral protein intake without supervised resistance training.	
				Standard care for OPBS.	
(20)	Participants’ BMI > 35 with obesity-related disease or BMI > 50 with obesity-related social or physical complications.	32 were allocated to intervention group (11 men and 20 women) and 28 were allocated to control group (7 men and 21 women).	Roux-en-Y gastric bypass.	Aerobic and strength training.	The supervised physical training intervention resulted in a 4.2-kg lower body weight in intervention group compared with control group at the study end. The high-density lipoprotein concentration was significantly higher in intervention group than in control group at the termination of the intervention, but this was not maintained at the 24-months examination.
				Standard care for OPBS.	
(19)	Participants’ BMI > 35 with obesity-related disease or BMI > 50 with obesity-related social or physical complications.	32 were allocated to intervention group (11 men and 20 women) and 28 were allocated to control group (7 men and 21 women).	Roux-en-Y gastric bypass.	Aerobic and strength training.	RYGB surgery alone has no effect on aerobic capacity, but decreases MS and improves physical function during the first 6 months following surgery. Supervised physical training following RYGB surgery is feasible and improves all three measures of physical capacity.
				Standard care for OPBS.	
(17)	Sixty white patients (15 male and 45 female), BMI ≥ 35 kg/m2, aged 20–50-year-olds, were recruited and underwent Roux-en-Y gastric bypass (n = 27) surgery or sleeve gastrectomy (n = 33).	20 were allocated to control group (4 men and 16 women) and 20 were allocated to aerobic group (5 men and 15 women) and 20 were allocated to aerobic and strength group (6 men and 14 women).	Roux-en-Y gastric bypass surgery or sleeve gastrectomy.	Standard care for OPBS.	Twelve weeks of aerobic or aerobic-strength exercise after bariatric surgery leads to greater weight and fat mass reduction. Doing exercise after surgery is more effective for improving the aerobic performance than surgery alone. Moreover, adding strength exercise to aerobic exercise after bariatric surgery reduces muscle mass loss and increases 1RM.
				Aerobic training.	
				Aerobic and strength training.	
(21)	BMI ≥ 35.5, exercising < 20min/day within the previous 3 months, undergone bariatric surgery at least 3 months earlier, and being 18–65 years old.	12 were allocated to control group (1 men and 11 women) and 21 were allocated to moderate-intensity aerobic group (2 men and 19 women).	Roux-en-Y gastric bypass and gastric banding.	Moderate-intensity aerobic exercise.	HVEP is feasible in about 50% of the patients and enhances physical fitness and reduces postprandial blood glucose in bariatric surgery patients.
				Standard care for OPBS.	

HR, heart rate; BMI, body mass index; GBS, gastric bypass surgery; OPBS, overweight patients after bariatric surgery; PA, physical activity; BS, bariatric surgery; TTM-based intervention, transtheoretical model–based exercise training program intervention; HDL, high-density lipoprotein; LDL, low-density lipoprotein; RM, repetition maximum; HVEP, high-intensity exercise program.

### 3.6 Synthesis of results

The studies can be broadly categorized into three main groups. The first group includes research focused on exercise training, investigating the effects of various regimens on OPBS (13–22). Auclair examined the impact of combining aerobic and strength training on cardiovascular function. Aguilar-Cordero explored the combined effects of aerobic, strength, and flexibility training. Ali assessed the benefits of home-based core exercises on stability and aerobic capacity. Castello-Simoes studied the effects of aerobic exercise on cardiovascular function in female OPBS patients. Hassannejad evaluated both the individual and combined contributions of aerobic and strength training on cardiovascular and physical function. Marc-Hernández investigated the influence of aerobic and strength training on cholesterol levels and body composition. Mundbjerg analyzed the combined effects of aerobic and strength training on cardiovascular and physical function. Shah researched the impact of moderate to high-intensity aerobic exercise on cardiovascular health, while Tardif explored the effects of aerobic and strength training on cholesterol levels. The second group focuses on nutrition, specifically the interaction between protein intake and physical activity in this population (23, 24). Jassil evaluated the influence of nutritional counseling combined with exercise guidance on physical mobility. Oppert studied the impact of exercise paired with various protein intake regimens on body composition.The third group centers on health management and the effects of different exercise prescriptions on OPBS (25–27). De Oliveira assessed the impact of a semi-supervised training program on physical function improvement. Olsén investigated the role of tailored exercise prescriptions in monitoring physical fitness, while Ren utilized a Transtheoretical Model-based exercise program to enhance physical function performance.

#### 3.6.1 Body mass index

**Figure 4:**
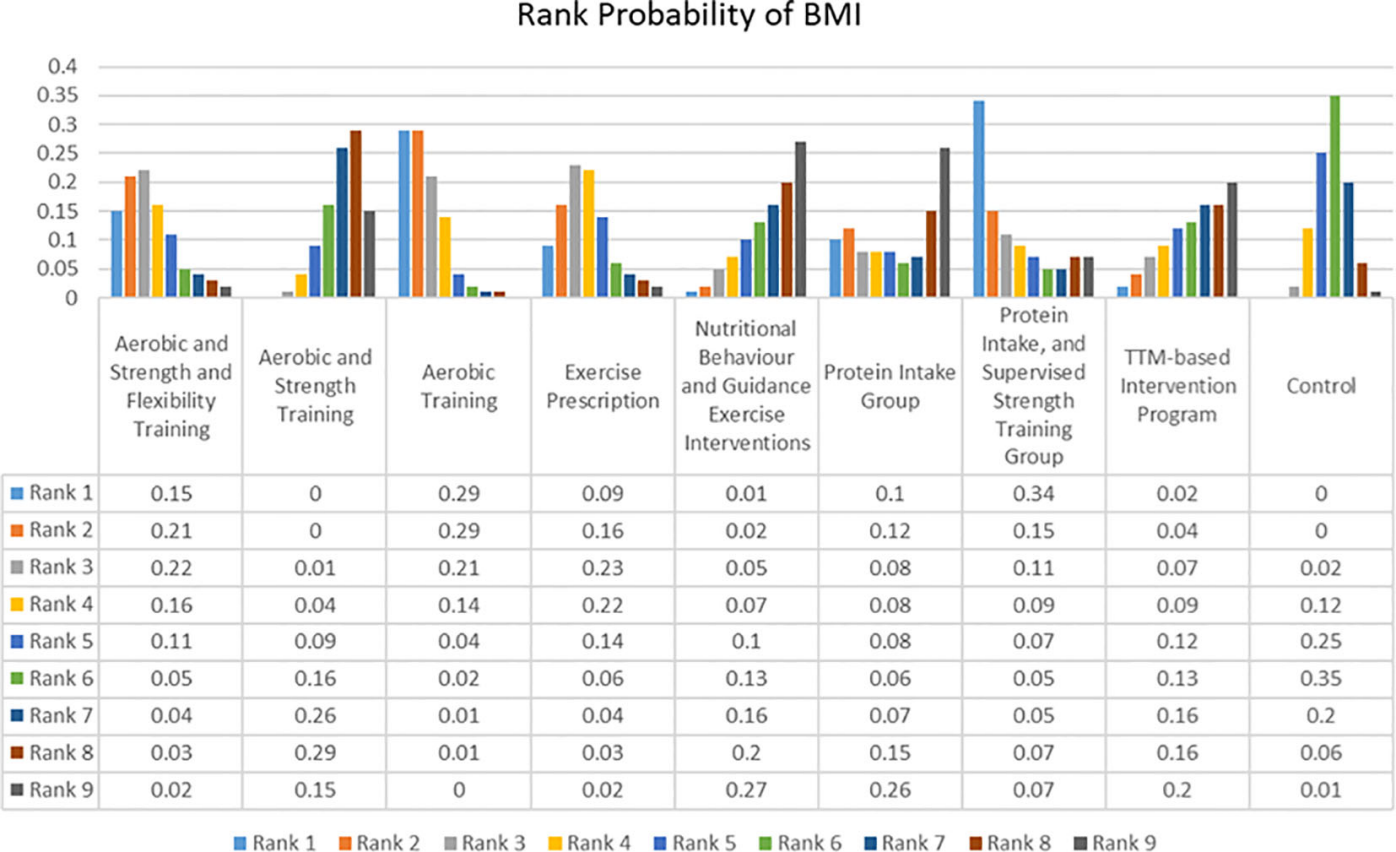
Rank probability of Body Mass Index (BMI).

The assessment of consistency models for different outcomes was presented in **Table 2** and the network of evidence for BMI was presented in **Figure 2A**. which had a closed loop with Random Effects Standard Deviation = 0.91 (0.06, 3.10) under the consistency model and 0.96 (0.06, 3.18) under the inconsistency model, after node split analysis, p=0.65. Therefore, the consistency model was selected for the analysis of the data. The rank probabilities of BMI were presented in **Figure 4**. The probability of being ranked as ‘Rank N’ was highest for NBGEI, at 0.27. While the probability of being ranked as ‘Rank N’ was lowest for AT, at 0. The highest probability of being ranked as Rank 1 was observed for PISST, at 0.34, while the lowest probability was observed for control group and AST, at 0.

**Table 2:** Assessment of consistency models for different outcomes.

Outcomes	Number of Closed Loops	Random Effects Standard Deviation in Consistency Model	Random Effects Standard Deviation in Inconsistency Model	P-Value in Node Split
**BMI**	1	0.91(0.06, 3.10)	0.96(0.06, 3.18)	0.65
**SBP**	0	4.77(0.34, 9.42)	4.74 (0.59, 9.38)	/
**PF**	1	0.44 (0.25, 0.89)	0.46 (0.26, 0.93)	0.84
**DBP**	0	2.23(0.01, 5.42)	2.28 0.17, 5.40)	/

BMI, body mass index; SBP, systolic blood pressure; PF, physical function; DBP, diastolic blood pressure.

#### 3.6.2 Blood pressure

**Figure 5:**
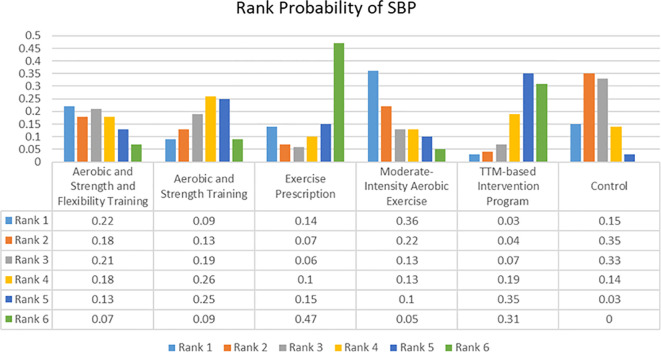
Rank probability of Systolic Blood Pressure (SBP).

The network of evidence for SBP was presented in **Figure 2D** which didn’t have closed loops with Random Effects Standard Deviation = 4.77(0.34, 9.42) under the consistency model and 4.74 (0.59, 9.38) under the inconsistency model. Therefore, the consistency model was selected for the analysis of the data. The rank probabilities of SBP were presented in **Figure 5**. The probability of being ranked as ‘Rank N’ was highest for EP at 0.47 and lowest for control group at 0. The probability of being ranked as ‘Rank 1’ was highest for MAE at 0.36 and lowest for TIP at 0.03.

**Figure 6:**
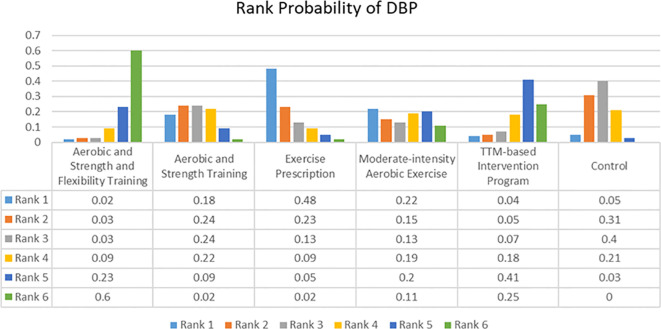
Rank probability of Diastolic Blood Pressure (DBP).

The network of evidence for DBP was presented in **Figure 2B** which didn’t have closed loops with Random Effects Standard Deviation = 2.23(0.01, 5.42) under the consistency model and 2.28 (0.17, 5.40) under the inconsistency model. Therefore, the consistency model was selected for the analysis of the data. The rank probabilities of DBP were presented in **Figure 6**. The probability of being ranked as ‘Rank N’ was highest for ASFT at 0.6, lowest for the control group at 0, and intermediate for TIP at 0.25. Meanwhile, EP was the highest probability of being ranked as ‘Rank 1’ at 0.48 and ASFT was the lowest probability of being ranked as ‘Rank 1’ at 0.02.

#### 3.6.3 Physical function

**Figure 7:**
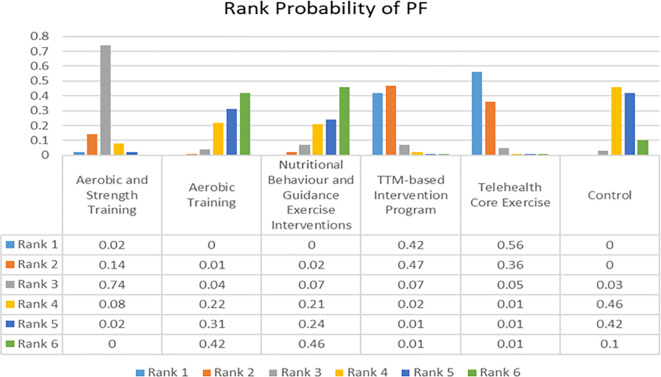
Rank probability of Physical Function (PF).

The network of evidence for PF was presented in **Figure 2C** which had a closed loop with Random Effects Standard Deviation = 0.44 (0.25, 0.89) under the consistency model and 0.46 (0.26, 0.93) under the inconsistency model, after node split analysis, p=0.84. Therefore, the consistency model was selected for the analysis of the data. The rank probabilities of PF were presented in **Figure 7**. The probability of being in the ‘Rank N’ category was highest for NBGEI at 0.46, while the lowest probability was observed for AST at 0. Meanwhile, the probability of being ranked as ‘Rank 1’ was highest for TCE at 0.56 and the probability of being ranked as ‘Rank 1’ was lowest for AT and Control and NBGEI at 0.

## 4 Discussion

This review aims to investigate the effects of different exercise interventions on the PF of OPGS. Based on the results, three key findings emerged. First, telehealth core exercise appeared to be the most effective intervention for improving PF. Second, nutritional behavior and guided exercise interventions, as well as protein intake interventions, were likely the most effective for improving BMI. Third, aerobic exercise combined with strength and flexibility training might be most effective for improving DBP, while exercise prescriptions might be more effective for improving SBP.

These findings may provide insight into the clinical research process. The primary finding of this study was that telehealth core exercise was most effective for enhancing PF. Previous research showed that balance training can significantly improve the balance abilities of OPBS (39). Additionally, studies indicated that whole-body vibration training can effectively reduce the risk of falls in obese patients (40). These findings were consistent with those of the current study. Based on previous research, it can be inferred that the improvement in PF was closely related to the strengthening of small muscle groups, core strength, and balance abilities (14). Conversely, the effects of AT and NBGEI on this outcome may be relatively unsatisfactory. Sole reliance on aerobic training may primarily improve cardiorespiratory function, but may not adequately train stabilizing muscle groups (41). Moreover, some studies shown that exercise superimposed on bariatric surgery increased connectivity between the hypothalamus and sensorial regions, and decreased default mode network and posterior salience network connectivity compared to bariatric surgery alone (42). It is also possible that different exercise interventions have different levels of impact on the connectivity between regions of the brain. This may be achieved by examining the underlying physiological mechanisms from a neuroscience perspective. Furthermore, in terms of improving BMI, nutritional behavior and guided exercise intervention and protein intake intervention were considered the most effective. Conversely, aerobic training group and protein intake and supervised strength training group were the least effective in improving BMI. However, other than the significant improvement in aerobic training and strength training group compared to aerobic training group, and the control group’s significant improvement over the aerobic training group, there were no significant differences among other interventions (95% CI did not include 0). This suggested that the improvement in BMI in most studies may be more dependent on the BS itself (16). This phenomenon can be attributed to the more restrained eating behaviors commonly observed in individuals who have undergone metabolic and bariatric surgery, as the surgical procedure induces significant anatomical and physiological changes. The findings of other studies that evaluated restrained eating behaviors in patients who had undergone metabolic and bariatric surgery also supported the results of the present study (43–48). In addition, BS can lead to inadequate protein intake and a state of negative nitrogen balance in obese patients, resulting in weight loss that is not closely related to the type of postoperative exercise intervention. This is crucial for further explaining the protein leverage hypothesis (49). Lastly, the results suggested that aerobic exercise combined with strength and flexibility training may be most effective for improving DBP, while exercise prescriptions may be more effective for improving SBP. At the same time, the other intervention groups showed better improvements in both systolic and diastolic blood pressure than the control group. These findings were consistent with previous research. For instance, studies found that combined aerobic and strength training had a significant effect on improving DBP in hypertensive patients (41). Coupled with prior animal studies, the potential mechanism for regulating BP may be that aerobic exercise prevented BP elevation through beneficial changes in insulin sensitivity and autonomic nervous system function, while resistance training prevented BP elevation through beneficial changes in vascular contraction regulation (50, 51). Additionally, hypertension is a common complication of obesity, and as such, its presence or absence will not be used as a variable affecting the results of the experiment (52). However, the precise mechanisms of such changes are not yet fully understood. It appears that the sensitivity of diastolic and systolic improvements to the same type of exercise intervention differs. It is possible that relatively gentle and individualised exercise interventions are more effective in reducing SBP, while more intense forms of exercise intervention may produce better improvements in DBP.

The primary strength of this study is its innovative use of network meta-analysis to systematically identify the most effective exercise interventions for enhancing PF in OPBS. By integrating direct and indirect evidence from multiple studies, network meta-analysis provides a comprehensive comparison of various exercise modalities, surpassing the limitations of traditional meta-analyses. This approach not only offers a clearer hierarchy of intervention efficacy but also equips clinicians and rehabilitation therapists with valuable insights for tailoring personalized treatment plans. The findings contribute significantly to the evidence base, supporting the development of more effective exercise prescriptions for improving PF in OPBS.

Nevertheless, this review has several limitations. Firstly, there was variation in the types of BS that subjects underwent, and few studies categorized them by type, which could introduce bias. Secondly, some trials included only women, limiting the ability to assess gender effects. Thirdly, the recent COVID-19 pandemic led some studies to implement remote monitoring interventions, which may obscure the effects of similar interventions in fixed-location settings and complicate the accurate assessment of adherence. Additionally, as a meta-analysis, it is challenging to control precisely for the timing of interventions across studies. Given the declining prevalence of COVID-19 globally, further RCTs are needed to explore the effects of different exercise interventions on PF in OPBS, focusing on the influence of surgical procedure, gender, and intervention delivery method.

Future research should prioritize core muscle training interventions for individuals with OPBS to better understand the role of stabilizing muscles in enhancing PF within this population. Additionally, further studies could investigate the effects of protein intake on body composition, aiming to clarify the unique digestive and metabolic characteristics associated with OPBS. It would also be beneficial for future research to explore the mechanisms by which various exercise modalities influence blood pressure changes in this group, providing a deeper understanding of how different training approaches can impact overall cardiovascular health.

## 5 Conclusion

Telehealth core exercises are the most effective intervention for improving PF in OPBS, likely due to their impact on strengthening small muscle groups. In terms of BMI reduction, the combination of nutritional behavior modifications, structured exercise programs, and increased protein intake yields the best results. For DBP reduction, a blend of aerobic exercise, strength training, and flexibility exercises proves most beneficial. Conversely, SBP is more effectively managed through personalized exercise prescriptions. Meanwhile, it is important to note that changes in BMI and blood pressure may be more attributable to the effects of surgery itself than to the specific postoperative exercise interventions. Thus, further rigorously designed RCTs are needed to draw definitive conclusions.

## Data Availability

The original contributions presented in the study are included in the article/**Supplementary Material**. Further inquiries can be directed to the corresponding authors.

## References

[1] WHO . Expert Committee on Physical Status: the Use and Interpretation of Anthropometry Physical status: the use and interpretation of anthropometry: report of a WHO expert committee. (United Kingdom: Clarivate). (1995).

[2] World Health, Organization . WHO | Obesity and overweight. (Geneva, Switzerland: WHO Press). (2013).

[3] Mantziari S Dayer A Duvoisin C Demartines N Allemann P Calmes JM . Long-Term Weight Loss, Metabolic Outcomes, and Quality of Life at 10Years After Roux-en-Y Gastric Bypass Are Independent of Patients' Age at Baseline. Obes Surg. (2020) 30:1181–8. doi: 10.1007/s11695-019-04181-z 32008256

[4] Ito MK Gonalves VSS Faria SLCM Moizé V Porporatti André Luís Guerra ENS . Effect of protein intake on the protein status and lean mass of post-bariatric surgery patients: a systematic review. Obes Surg. (2017) 27:502–12. doi: 10.1007/s11695-016-2453-0 27844254

[5] Haghighat N Ashtary-Larky D Bagheri R Aghakhani L Asbaghi O Amini M . Preservation of fat-free mass in the first year after bariatric surgery: a systematic review and meta-analysis of 122 studies and 10,758 participants. Surg Obes Related Dis. (2022) 18:964–82. doi: 10.1016/j.soard.2022.02.022 35581110

[6] Jung HN Kim SO Jung CH Lee WJ Kim MJ Cho YK . Preserved muscle strength despite muscle mass loss after bariatric metabolic surgery: a systematic review and meta-analysis. Obes Surg. (2023) 33:3422–30. doi: 10.1007/s11695-023-06796-9 PMC10602996 37728838

[7] Nuijten MAH Monpellier VM Eijsvogels T Janssen IMC Hazebroek EJ Hopman MTE . Rate and determinants of excessive fat-free mass loss after bariatric surgery. Obes Surg. (2020) 30:3119–26. doi: 10.1007/s11695-020-04654-6 PMC7305251 32415634

[8] Nuijten MAH Eijsvogels TMH Monpellier VM Janssen IMC Hazebroek EJ Hopman MTE . The magnitude and progress of lean body mass, fat-free mass, and skeletal muscle mass loss following bariatric surgery: A systematic review and meta-analysis. Obes Rev. (2022) 23. doi: 10.1111/obr.13370 PMC9285034 34664391

[9] Caspersen CJ Christenson PGM . Physical activity, exercise, and physical fitness: definitions and distinctions for health-related research. Public Health Rep. (1985) 100:126–31. PMC1424733 3920711

[10] Wen CP Wai JPM Tsai MK Yang YiC Cheng TYD Lee MC . Minimum amount of physical activity for reduced mortality and extended life expectancy: a prospective cohort study. Lancet. (2011) 378:1244–53. doi: 10.1016/S0140-6736(11)60749-6 21846575

[11] Elley CR Arroll B . Refining the exercise prescription for hypertension. LANCET. (2005) 366:1248–9. doi: 10.1016/S0140-6736(05)67507-1 16214585

[12] Holme I Anderssen SA . Increases in physical activity is as important as smoking cessation for reduction in total mortality in elderly men: 12 years of follow-up of the Oslo II study. Br J Sports Med. (2015) 49:1–6. doi: 10.1136/bjsports-2014-094522 25977572

[13] Auclair A Harvey J Leclerc J Piché M-E Poirier P . Determinants of cardiorespiratory fitness following bariatric surgery: insights from a randomized controlled trial of a supervised training program. Can J Cardiol. (2020) 37(2):251–9. doi: 10.1016/j.cjca.2020.03.032 32738206

[14] Aguilar-Cordero MJ Rodríguez-Blanque R Hernández CL Inzunza-Noack J Sánchez-García JC Noack-Segovia J . Physical exercise to improve functional capacity: randomized clinical trial in bariatric surgery population. J Clin Med. (2022) 11:4621. doi: 10.3390/jcm11154621 PMC9369494 35956235

[15] Ali OI Abdelraouf OR El-Gendy AM Abdelgalil AA Abdelaal AK Dahlawi HA . Efficacy of telehealth core exercises during COVID-19 after bariatric surgery: a randomized controlled trial. Eur J Phys Rehabil Med. (2022) 58:845–52. doi: 10.23736/s1973-9087.22.07457-3 PMC10077962 35904308

[16] Castello-Simoes V Simoes RP Beltrame T Bassi D Catai AM Arena R . Effects of aerobic exercise training on variability and heart rate kinetic during submaximal exercise after gastric bypass surgery - a randomized controlled trial. Disability Rehabil. (2013) 35:334–42. doi: 10.3109/09638288.2012.694575 22725971

[17] Hassannejad A Khalaj A Mansournia MA Tabesh MR Alizadeh Z . The effect of aerobic or aerobic-strength exercise on body composition and functional capacity in patients with BMI ≥35 after bariatric surgery: a randomized control trial. Obes Surg. (2017) 27:2792–801. doi: 10.1007/s11695-017-2717-3 28527156

[18] Marc-Hernández A Ruiz-Tovar J Aracil A Guillén S Moya-Ramón M . Effects of a high-intensity exercise program on weight regain and cardio-metabolic profile after 3 years of bariatric surgery: A randomized trial. Sci Rep. (2020) 10. doi: 10.1038/s41598-020-60044-z PMC7033151 32080310

[19] Mundbjerg LH Stolberg CR Bladbjerg EM Funch-Jensen P Juhl CB Gram B . Effects of 6 months supervised physical training on muscle strength and aerobic capacity in patients undergoing Roux-en-Y gastric bypass surgery: a randomized controlled trial. Clin Obes. (2018) 8:227–35. doi: 10.1111/cob.12256 29896844

[20] Mundbjerg LH Stolberg CR Cecere S Bladbjerg EM Funch-Jensen P Gram B . Supervised physical training improves weight loss after roux-en-Y gastric bypass surgery: A randomized controlled trial. Obesity. (2018) 26:828–37. doi: 10.1002/oby.22143 29566463

[21] Shah M Snell PG Rao S Adams-Huet B Quittner C Livingston EH . High-volume exercise program in obese bariatric surgery patients: A randomized, controlled trial. Obesity. (2011) 19:1826–34. doi: 10.1038/oby.2011.172 21681226

[22] Tardif I Auclair A Piché ME Biertho L Marceau S Hould FS . Impact of a 12-week randomized exercise training program on lipid profile in severely obese patients following bariatric surgery. Obes Surg. (2020) 30:3030–6. doi: 10.1007/s11695-020-04647-5 32367175

[23] Jassil FC Carnemolla A Kingett H Doyle J Kirk A Lewis N . Impact of nutritional-behavioral and supervised exercise intervention following bariatric surgery: The BARI-LIFESTYLE randomized controlled trial. Obesity. (2023) 31:2031–42. doi: 10.1002/oby.23814 37415246

[24] Oppert JM Bellicha A Roda C Bouillot JL Torcivia A Clement K . Resistance training and protein supplementation increase strength after bariatric surgery: A randomized controlled trial. Obesity. (2018) 26:1709–20. doi: 10.1002/oby.22317 30358153

[25] de Oliveira GN Goessler KF Santos JVP de Lima AP Genário R Merege CAA . Home-based exercise training during COVID-19 pandemic in post-bariatric patients: a randomized controlled trial. Obes Surg. (2021) 31:5071–8. doi: 10.1007/s11695-021-05621-5 PMC8349309 34365592

[26] Olsén MF Wiklund M Sandberg E Lundqvist S Dean E . Long-term effects of physical activity prescription after bariatric surgery: A randomized controlled trial. Physiotherapy Theory Pract. (2022) 38:1591–601. doi: 10.1080/09593985.2021.1885087 33576284

[27] Ren ZQ Zhu HF Zhang TZ Hua HX Zhao K Yang NL . Effects of a 12-week transtheoretical model-based exercise training program in chinese postoperative bariatric patients: a randomized controlled trial. Obes Surg. (2021) 31:4436–51. doi: 10.1007/s11695-021-05607-3 34373988

[28] Higgins J Green SR . Cochrane Handbook for Systematic Review of InterventionsVersion 5.1.0. (Chichester, UK: Wiley Online Library). (2011).

[29] Moher D Altman DG PRISMA Group . Preferred reporting items for systematic reviews and meta-analyses: the PRISMA statement. PloS Med. (2009) 339:264-W64. doi: 10.7326/0003-4819-151-4-200908180-00135 PMC3090117 21603045

[30] Borg G . ATS statement: guidelines for the six-minute walk test. Am J Respir Crit Care Med. (2016) 193:1185–5. doi: 10.1164/rccm.19310erratum 27174486

[31] Stroup DF Berlin JA Morton SC Olkin I Williamson GD Rennie D . Meta-analysis of observational studies in epidemiology - A proposal for reporting. Jama-Journal Am Med Assoc. (2000) 283:2008–12. doi: 10.1001/jama.283.15.2008 10789670

[32] Dias S Welton NJ Caldwell DM Ades AE . Checking consistency in mixed treatment comparison meta-analysis. Stat Med. (2010) 29:932–44. doi: 10.1002/sim.3767 20213715

[33] Rouse B Chaimani A Li TJ . Network meta-analysis: an introduction for clinicians. Internal Emergency Med. (2017) 12:103–11. doi: 10.1007/s11739-016-1583-7 PMC5247317 27913917

[34] Lu GB Ades AE . Assessing evidence inconsistency in mixed treatment comparisons. J Am Stat Assoc. (2006) 101:447–59. doi: 10.1198/016214505000001302

[35] Dias S Sutton AJ Ades AE Welton NJ . Evidence synthesis for decision making 2: A generalized linear modeling framework for pairwise and network meta-analysis of randomized controlled trials. Med Decision Making. (2013) 33:607–17. doi: 10.1177/0272989x12458724 PMC3704203 23104435

[36] Catalá-López F Tobías A Cameron C Moher D Hutton B . Network meta-analysis for comparing treatment effects of multiple interventions: an introduction. Rheumatol Int. (2014) 34:1489–96. doi: 10.1007/s00296-014-2994-2 24691560

[37] Armijo-Olivo S Stiles CR Hagen NA Biondo PD Cummings GG . Assessment of study quality for systematic reviews: a comparison of the Cochrane Collaboration Risk of Bias Tool and the Effective Public Health Practice Project Quality Assessment Tool: methodological research. J Eval Clin Pract. (2012) 18:12–8. doi: 10.1111/j.1365-2753.2010.01516.x 20698919

[38] Salanti G Ades AE Ioannidis JPA . Graphical methods and numerical summaries for presenting results from multiple-treatment meta-analysis: an overview and tutorial. J Clin Epidemiol. (2011) 64:163–71. doi: 10.1016/j.jclinepi.2010.03.016 20688472

[39] Rojhani-Shirazi Z Mansoriyan SA Hosseini SV . The effect of balance training on clinical balance performance in obese patients aged 20-50 years old undergoing sleeve gastrectomy. Eur Surgery-Acta Chirurgica Austriaca. (2016) 48:105–9. doi: 10.1007/s10353-015-0379-8

[40] Yang F Munoz J Han LZ Yang F . Effects of vibration training in reducing risk of slip-related falls among young adults with obesity. J Biomechanics. (2017) 57:87–93. doi: 10.1016/j.jbiomech.2017.03.024 28431747

[41] Baak MA Pramono A Battista F Beaulieu K Blundell JE Busetto L . Effect of different types of regular exercise on physical fitness in adults with overweight or obesity: Systematic review and meta-analyses. Obes Rev. (2021) 22(S4):e13239. doi: 10.1111/obr.13239 PMC8365680 33939229

[42] Merege CAA Gil SS Kirwan JP Murai IH Dantas WS Nucci MP . Exercise modifies hypothalamic connectivity and brain functional networks in women after bariatric surgery: a randomized clinical trial. Int J Obes. (2023) 47:165–74. doi: 10.1038/s41366-022-01251-8 PMC10134041 36585494

[43] Batsis JA Clark MM Grothe K Lopez-Jimenez F Collazo-Clavell ML Somers VK . Self-efficacy after bariatric surgery for obesity. A population-based cohort study. Appetite. (2009) 52:637–45. doi: 10.1016/j.appet.2009.02.017 19501761

[44] Ashford S Edmunds J French DP . What is the best way to change self-efficacy to promote lifestyle and recreational physical activity? A systematic review with meta-analysis. Br J Health Psychol. (2010) 15:265–88. doi: 10.1348/135910709x461752 19586583

[45] Novelli IR Fonseca LG Gomes DL Dutra ES de Carvalho KMB . Emotional eating behavior hinders body weight loss in women after Roux-en-Y gastric bypass surgery. Nutrition. (2018) 49:13–6. doi: 10.1016/j.nut.2017.11.017 29571605

[46] Subramaniam K Low WY Lau PC Chin KF Chinna K Kosai NR . Eating behaviour predicts weight loss six months after bariatric surgery: A longitudinal study. Nutrients. (2018) 10. doi: 10.3390/nu10111616 PMC6266615 30400129

[47] Konings G Drukker M Mulkens S Severeijns R van Os J Ponds R . Postsurgical compliance and eating behavior 5 years after surgery. Bariatric Surg Pract Patient Care. (2020) 15:148–54. doi: 10.1089/bari.2019.0049

[48] Al-Najim W Docherty NG le Roux CW . Food intake and eating behavior after bariatric surgery. Physiol Rev. (2018) 98:1113–41. doi: 10.1152/physrev.00021.2017 29717927

[49] Diaz KM Shimbo D . Physical activity and the prevention of hypertension. Curr Hypertension Rep. (2013) 15:659–68. doi: 10.1007/s11906-013-0386-8 PMC3901083 24052212

[50] Moraes-Silva IC Mostarda C Moreira ED Silva KAS dos Santos F de Angelis K . Preventive role of exercise training in autonomic, hemodynamic, and metabolic parameters in rats under high risk of metabolic syndrome development. J Appl Physiol. (2013) 114:786–91. doi: 10.1152/japplphysiol.00586.2012 23329818

[51] de Araujo AJ dos Santos ACV Souza KD Aires MB Santana-Filho VJ Fioretto ET . Resistance training controls arterial blood pressure in rats with L-NAME-induced hypertension. Arquivos Brasileiros Cardiologia. (2013) 100:339–46. doi: 10.5935/abc.20130051 23545992

[52] Cowan GSM Buffington CK . Significant changes in blood pressure, glucose, and lipids with gastric bypass surgery. World J OF Surg. (1998) 22:987–92. doi: 10.1007/s002689900504 9717426

[53] Batterham RL National Institute for Health and Care Excellence . Obesity-Identification, assessment and management of overweight and obesity in childen, young people an adults. (London, United Kingdom: National Institute for Health and Care Excellence (NICE)). (2014). p. CG43. 25535639

